# Genetic Correlations Among Corneal Biophysical Parameters and Anthropometric Traits

**DOI:** 10.1167/tvst.12.8.8

**Published:** 2023-08-10

**Authors:** Henry C. Cousins, Clara C. Cousins, Girish Valluru, Russ B. Altman, Yutao Liu, Louis R. Pasquale, Sumayya Ahmad

**Affiliations:** 1 Department of Biomedical Data Science, Stanford University School of Medicine, Stanford, CA, USA; 2 David Geffen School of Medicine, University of California, Los Angeles, Los Angeles, CA, USA; 3 Department of Ophthalmology, New York Eye and Ear Infirmary of Mount Sinai, Icahn School of Medicine at Mount Sinai, New York, NY, USA; 4 Department of Cellular Biology and Anatomy, Augusta University, Augusta, GA, USA

**Keywords:** corneal topography, genetic diseases, genetic correlations

## Abstract

**Purpose:**

The genetic architecture of corneal dysfunction remains poorly understood. Epidemiological and clinical evidence suggests a relationship between corneal structural features and anthropometric measures. We used global and local genetic similarity analysis to identify genomic features that may underlie structural corneal dysfunction.

**Methods:**

We assembled genome-wide association study summary statistics for corneal features (central corneal thickness, corneal hysteresis [CH], corneal resistance factor [CRF], and the 3 mm index of keratometry) and anthropometric traits (body mass index, weight, and height) in Europeans. We calculated global genetic correlations (*r*_g_) between traits using linkage disequilibrium (LD) score regression and local genetic covariance using ρ-HESS, which partitions the genome and performs regression with LD regions. Finally, we identified genes located within regions of significant genetic covariance and analyzed patterns of tissue expression and pathway enrichment.

**Results:**

Global LD score regression revealed significant negative correlations between height and both CH (*r*_g_ = −0.12; *P* = 2.0 × 10^−7^) and CRF (*r*_g_ = −0.11; *P* = 6.9 × 10^−7^). Local analysis revealed 68 genomic regions exhibiting significant local genetic covariance between CRF and height, containing 2874 unique genes. Pathway analysis of genes in regions with significant local *r*_g_ revealed enrichment among signaling pathways with known keratoconus associations, including cadherin and Wnt signaling, as well as enrichment of genes modulated by copper and zinc ions.

**Conclusions:**

Corneal biophysical parameters and height share a common genomic architecture, which may facilitate identification of disease-associated genes and therapies for corneal ectasias.

**Translational Relevance:**

Local genetic covariance analysis enables the identification of associated genes and therapeutic targets for corneal ectatic disease.

## Introduction

The causes and consequences of corneal biomechanical dysfunction are complex. Although the biophysical features underlying corneal shape contribute directly to ocular diseases such as keratoconus, they also demonstrate associations with systemic disorders, particularly those of the connective tissue.^1^ For instance, Marfan syndrome, a systemic disorder of impaired microfibril formation classically resulting in elongated bone structures, is associated with reduced corneal curvature.^2^^–^^4^ Ehlers-Danlos syndrome and Williams syndrome, both of which result from defective collagen and elastin synthesis and are also associated with taller stature, contribute to thinner and steeper corneal contours.^5^^–^^7^ In contrast, Turner syndrome and Down syndrome, both canonically associated with lower body heights, are associated with specific corneal phenotypes such as changes in central corneal thickness (CCT), compared to healthy controls.^8^^–^^10^ More broadly, recent population-scale evidence suggests an association between reduced corneal refractive power, indicative of corneal flatness, and increased body height.^11^ Such conditions collectively demonstrate a varied genetic landscape encompassing both autosomal and sex-chromosomal defects, suggesting the existence of an underlying genomic relationship between anthropometric features and corneal biophysical traits.^12^

Currently, the specific genomic drivers of corneal biomechanical dysfunction remain poorly understood, despite evidence that genetic polymorphisms explain a large proportion of such phenotypic variation.^13^^–^^15^ Keratoconus itself is believed to share a genomic underpinning with systemic connective tissue disorders. One genome-wide association study (GWAS) identified significant keratoconus associations for 12 novel loci, implicating both collagen matrix production and cell differentiation pathways.^15^ Another GWAS in 2021 identified 32 genetic variants associated with corneal curvature, most notably Wnt and fibroblast growth factor signaling.^16^ In 2022, a GWAS relying on biophysical endophenotypes as surrogates of keratoconus identified 150 novel loci and similarly implicated pathways involved in connective tissue synthesis.^17^ Furthermore, corneal curvature, anterior chamber depth, and axial length are themselves highly heritable,^18^ with corneal curvature and axial length demonstrating significant genetic correlation.^19^

Although association studies have identified some genomic variants associated with keratoconus, traditional GWAS are limited in their ability to resolve common, low-effect variants that likely underlie a large proportion of corneal ectasia heritability.^13^^,^^20^^,^^21^ Such variants may be studied indirectly through genetic correlation analysis, which compares correlations between measured variant effects for distinct phenotypes.^22^ Such analysis can be performed globally, comparing correlation among all measured variants to assess the genomic similarity of given phenotypes, or locally, measuring region-specific correlations to implicate specific patterns of variation shared in both conditions. To clarify the shared genomic underpinnings of corneal ectasia and systemic connective-tissue development, we performed a genetic correlation analysis of corneal biophysical features and anthropometric features.

## Methods

### Ethical Statement

Because summary-level genetic data used in this study are publicly available and deidentified, institutional review board review for this project was not required. Bulk corneal gene expression data were made available from a previous study, which was approved by the institutional review board at Augusta University.^23^

### GWAS Datasets

We obtained summary statistics for publicly available GWAS of keratoconus-related and anthropometric traits, including CCT, corneal hysteresis (CH), corneal resistance factor (CRF), 3 mm index of keratometry result, body mass index (BMI), weight, and height. All GWAS studies considered in the analysis are summarized in Table 1, and the summary data, as well as details on variant calling and preprocessing, are available at the respective citations, as well as via the Neal Lab's data repository for these GWAS studies, available at http://www.nealelab.is/uk-biobank/.

**Table 1.** Summary of Traits Analyzed in Genome-Wide Association Studies

Trait	Description	Sample Size	GWAS-Explained Heritability (SE)	Reference (PMID)
CCT	Maximum thickness of cornea (higher value represents thicker tissue)	17,803	0.32 (0.04)	Iglesias et al. 2018 (29760442)^*^
CH of the right eye	Viscoelastic damping of cornea (higher value represents greater damping strength)	76,630	0.20 (0.01)	Bycroft et al. 2018 (30305743)^†^
CRF of the right eye	Corneal elasticity (relatively independent of pressure; higher value represents greater elasticity)	76,630	0.25 (0.02)	Bycroft et al. 2018 (30305743)^†^
3 mm index of keratometry result of the right eye	Corneal curvature (higher value represents greater curvature)	75,410	0.0049 (0.01)	Bycroft et al. 2018 (30305743)^†^
BMI	Height in meters divided by square of weight in kilograms	339,224	0.14 (0.01)	Bycroft et al. 2018 (30305743)^†^
Weight	In kilograms	360,116	0.26 (0.01)	Bycroft et al. 2018 (30305743)^†^
Height (UK Biobank)	In meters	360,388	0.46 (0.01)	Bycroft et al. 2018 (30305743)^†^

PMID, PubMed unique identifier.

* Iglesias AI, Mishra A, Vitart V, et al. Cross-ancestry genome-wide association analysis of corneal thickness strengthens link between complex and Mendelian eye diseases [published correction appears in *Nat Commun*. 2019 Jan 8;10(1):155]. *Nat Commun*. 2018;9(1):1864. doi:10.1038/s41467-018-03646-6.

† Bycroft C, Freeman C, Petkova D, et al. The UK Biobank resource with deep phenotyping and genomic data. *Nature*. 2018;562(7726):203-209. doi:10.1038/s41586-018-0579-z.

Both corneal biophysical parameters and anthropometric traits showed significant heritability explained in their respective GWAS data, with explained heritability proportions ranging from 0.14 (BMI) to 0.46 (height). The 3 mm index of keratometry result was an outlier, with an explained heritability of 0.0049.

### Global Genetic Correlations

Genetic correlations occur in the presence of shared single nucleotide polymorphism (SNP) heritability. SNP heritability is the proportion of variability in a trait that is due to genetic contributions, and it can be estimated by classical twin studies or from regressing test statistics from SNPs in a GWAS across linkage disequilibrium (LD) blocks.^24^ As implemented in the LDSC package (https://github.com/bulik/ldsc),^24^^,^^25^ we calculated GWAS-explained heritability using all SNPs included in the GWAS. We calculated genome-wide genetic correlation as the covariance of two traits divided by the product of the traits’ heritabilities. Because LD blocks vary with ancestry, we confined our analysis to GWAS with participants of European ancestry. We used genome-wide LD score regression as implemented in the LDSC package. The Bonferroni correction (α = 0.05, n = 21) was applied to account for multiple testing. Power calculations for all pairwise analyses are provided in Supplementary Table S1.

### Local Genetic Correlations

We used ρ-HESS (https://huwenboshi.github.io/hess/)^26^ to estimate the shared heritability of two traits across partitions of the genome. This method applies a fixed effect model to assess the covariance to the combined effects of SNPs on the two traits. In local genetic covariance analysis, the genome is partitioned into approximately independent LD regions with an average width of 1.6 Mb. This results in 1703 regions across all chromosomes, excluding the sex chromosomes. These regions were the same as the ones implemented by default in ρ-HESS as we had no rationale to prioritize different genome partitions. We conducted local genetic covariance analyses for CRF and height given their high global genetic correlation. We also stratified CRF by sex and performed global genetic correlation analysis with the other corneal and anthropometric traits.

We prioritized individual genes for downstream analysis by the significance of local covariance between CRF and height. CRF was selected as the endophenotype of choice for this analysis because it had the largest set of significant SNPs of the biophysical traits investigated.^17^ Specifically, we identified all unique genes in the GRCh37 reference assembly located within regions exhibiting significant local *r*_g_ after Bonferroni correction (α = 0.05, n = 1703). Genome mapping was performed using PyEnsembl (release 97). A full list of the genes is provided as supplementary data.

### Tissue Expression Analysis

We performed single-cell expression analysis of genes in regions of high local covariance using an atlas of gene expression in 19 cell types of the anterior chamber of healthy human eyes.^27^ These cell types included corneal epithelium, B-cells, macrophages, natural killer cells, mast cells, Schwalbe cells, fibroblasts, collector channel cells, vascular endothelium, Schlemm canal cells, juxtacanalicular tissue cells, beam cells (types A and B), ciliary muscle cells, pericytes, melanocytes, myelinating and non-myelinating Schwann cells, and neurons. In addition to single-cell analysis of healthy tissue, we also compared the expression of genes in regions of high local covariance using bulk corneal tissue from 10 keratoconus patients and 8 healthy controls.^23^ Comparisons were performed using Mann-Whitney U tests, with a two-sided significance level of 0.05.

### Functional Gene Annotation

We compared the overlap of gene candidates in regions of significant local covariance with genes associated with either keratoconus or body height in the existing literature. Literature-based gene-phenotype associations were obtained from the DisGeNET ontology, which integrates clinical associations, experimental evidence, and computational predictions to produce a confidence score for gene-phenotype associations.^28^

Functional pathway enrichment among gene candidates was measured using gene set enrichment analysis.^29^ Analyses were performed in WebGestalt, using PANTHER pathway gene sets to evaluate pathway involvement and DrugBank drug-target gene sets for drug associations, in both cases using the default running parameters.^30^^–^^32^

## Results

### Global Genetic Correlations Between Traits

Both corneal biophysical parameters and anthropometric traits showed highly significant correlations among themselves, as expected. CH was highly positively correlated with CCT (*r*_g_ = 0.64; standard error (SE) = 0.06; *P* = 3.0e-24), as was CH with CRF (*r*_g_ = 0.88; SE = 0.01; *P* < 1e-100) and CCT with CRF (*r*_g_ = 0.71; SE = 0.06; *P* = 8.7e-36). Furthermore, there were extreme positive correlations between weight and both height (*r*_g_ = 0.43; SE = 0.02; *P* < 1e-100) and BMI (*r*_g_ = 0.81; SE = 0.02; *P* < 1e-100), as well as a weaker negative correlation between height and BMI (*r*_g_ = −0.09; SE = 0.02; *P* = 6.3e-6), consistent with previous evidence.^25^

Genetic correlation analysis revealed significant inverse global associations between corneal biophysical features and anthropometric traits (Table 2). Specifically, we observed significant negative genetic correlations between CRF and height (*r*_g_ = −0.11; SE = 0.02; *P* = 6.9e-7), as well as between CH and height (*r*_g_ = −0.12; SE = 0.02; *P* = 2.0e-7). No other pairwise correlations reached significance. The sex-stratified CRF and height genetic correlations (Supplementary Table S2) were similar (females only: *r*_g_ = −0.11; SE = 0.02; *P* = 6.0e-6; males only: *r*_g_ = −0.11; SE = 0.03; *P* = 6.0e-4).

**Table 2.** Genetic Correlations Among Keratoconus-Related and Anthropometric Traits^*^

	CH	CRF	3mmK	BMI	Weight	Height
CCT	**0.64 (0.06)**	**0.71 (0.06)**	−0.37 (0.36)	0.03 (0.04)	−0.01 (0.03)	−0.03 (0.03)
	***P* = 3.0e-24**	***P* = 8.7e-36**	*P* = 0.29	*P* = 0.38	*P* = 0.62	*P* = 0.34
CH	–	**0.88 (0.01)**	−0.26 (0.24)	0.03 (0.03)	−0.002 (0.02)	−**0.12 (0.02)**
		***P* < 1e-100**	*P* = 0.29	*P* = 0.38	*P* = 0.93	***P* = 2.0e-7**
CRF	–	–	−0.22 (0.22)	0.02 (0.03)	−0.01 (0.02)	−**0.11 (0.02)**
			*P* = 0.33	*P* = 0.54	*P* = 0.58	***P* = 6.9e-7**
3mmK	–	–	–	0.15 (0.17)	0.23 (0.20)	0.14 (0.15)
				*P* = 0.37	*P* = 0.25	*P* = 0.32
BMI	–	–	–	–	**0.81 (0.02)**	−**0.09 (0.02)**
					***P* < 1e-100**	***P* = 6.3e-6**
Weight	–	–	–	–	–	**0.43 (0.02)**
						***P* < 1e-100**

3mmK, 3 mm index of keratometry result.

* Data are presented as global r_g_ (SE); *P* value. Bold text indicates statistical significance after Bonferroni correction.

### Local Genetic Correlations for CRF and Height

We next searched for regions of significant local genetic covariance between height and corneal biophysical parameters, which represent drivers of the global genetic correlation between the two phenotypes. Given the high correlation between CRF and CH (*r*_g_ = 0.88) and the higher heritability for CRF, we restricted our analysis to the local drivers of the CRF-height genetic association. After adjusting for multiple hypothesis testing, 68 genomic regions showed significant local genetic covariance (Table 3; Supplementary Table S3). The regions had an average length of 1.7 Mb (standard deviation [SD] = 0.75 Mb) and contained an average of 2930 (SD = 1160) unique SNPs and 43 (SD = 38) unique genes per region. Although the global correlation was negative between CRF and height, we observed mixed directionality of covariance at the local level, with 51 significant negative and 17 significant positive covariances.

**Table 3.** Ten Regions of Strongest Local Genetic Covariance Between Corneal Resistance Factor and Height

Chr.	Region Length (kb)	Number of SNPs in the Region	Number of Genes in the Region	Local r_g_	Local r_g_ Variance	Local r_g_ SE	Local r_g_ *P* Value
3	1385	2119	23	1.65e-3	3.04e-8	1.74e-4	2.8e-21
5	1360	2727	11	−7.63e-4	7.17e-9	8.47e-5	2.0e-19
3	1331	2551	21	−1.10e-3	1.69e-8	1.30e-4	3.0e-17
21	628	1721	25	5.93e-4	5.21e-9	7.22e-5	2.2e-16
1	1938	2633	29	−6.27e-4	6.72e-9	8.20e-5	2.0e-14
2	1312	2947	19	5.31e-4	4.93e-9	7.02e-5	4.2e-14
17	1137	2304	33	−4.13e-4	3.14e-9	5.60e-5	1.6e-13
17	2068	2459	103	−6.30e-4	7.82e-9	8.84e-5	1.0e-12
15	1923	3143	33	−5.85e-4	7.01e-9	8.37e-5	2.8e-12
15	916	2295	23	−7.27e-4	1.12e-8	1.06e-4	6.7e-12

### Tissue Expression of Correlated Genes

**Figure 1.**
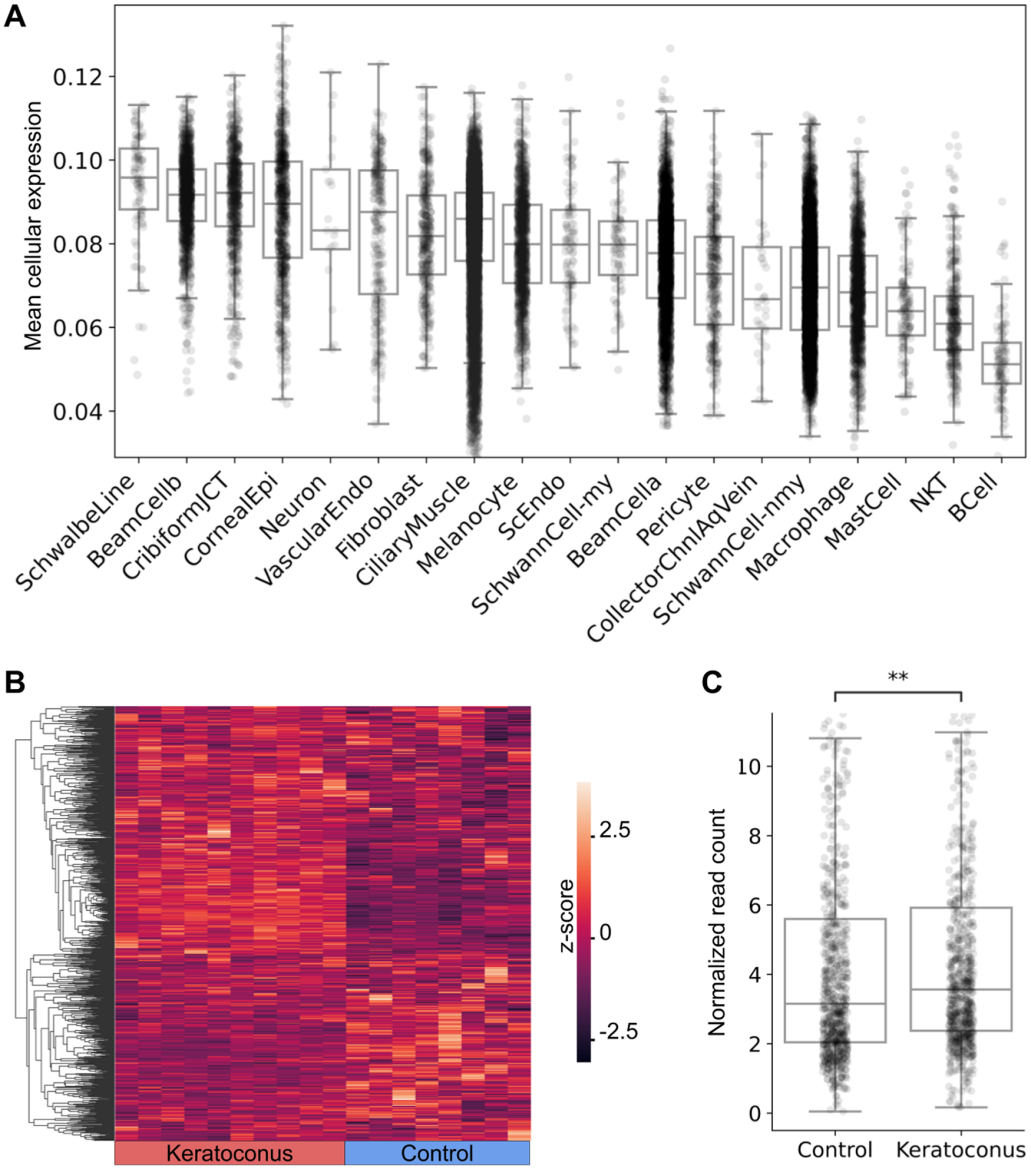
Expression in human ocular tissue of genes in regions of significant genetic covariance. (**A**) Distribution of single-cell expression levels in ocular cell types for genes in regions of significant covariance reveals high expression in cornea-related cell types, including Schwalbe cells and corneal epithelial cells. *BeamCella*, type A beam cells; *BeamCellb*, type B beam cells; *CollectorChnIAqVein*, collector channel cells; *CribiformJCT*, juxtacanalicular tissue cells; *NKT*, natural killer cells; *ScEndo*, Schlemm canal cells; *SchwannCell-my*, myelinating Schwann cells; *SchwannCell-nmy*, nonmyelinating Schwann cells. (**B**, **C**) Overall expression levels of genes in regions of high covariance were significantly greater in bulk corneal tissue from keratoconus patients than in corneal tissue from controls.

We identified 2874 unique genes in regions of significant local covariance. To determine whether these genes could represent shared genetic drivers between corneal dysfunction and height, we evaluated tissue expression patterns among genes in highly correlated regions (Fig. 1A) using an ocular anterior chamber single-cell gene expression dataset. Among the 19 cell types investigated, genes in regions of high covariance showed the greatest mean expression in corneal endothelial (Schwalbe) cells and the lowest in B lymphocytes. Mean expression in Schwalbe corneal endothelial cells of genes in regions with high covariance was significantly higher than in all other cell types (*P* ≤ 0.035 in all cases). Furthermore, expression in corneal epithelial cells was also high, being significantly greater (*P* ≤ 0.001 in all cases) than that in all but four other cell types (Schwalbe, Type B beam, juxtacanalicular tissue, and neuronal cells). We finally compared expression levels of genes in regions of high local covariance in corneal tissue between patients with keratoconus and healthy controls, observing that genes in regions of high genetic covariance showed 13% higher median expression levels in corneas with keratoconus than in healthy corneas (*P* = 0.003; Figs. 1B, 1C).

### Functional Pathway Enrichment Among Correlated Genes

We also evaluated whether genes in regions of high local genetic covariance included genes with known associations with both keratoconus and body height in a literature-based ontology of gene-phenotype associations.^28^ Among the 2874 genes contained in locally correlated regions, 18 had known associations with keratoconus, whereas 153 had known associations with body height, and three genes, *NOX4, FNDC3B,* and *ADAMTS17*, had known associations with both.

**Figure 2.**
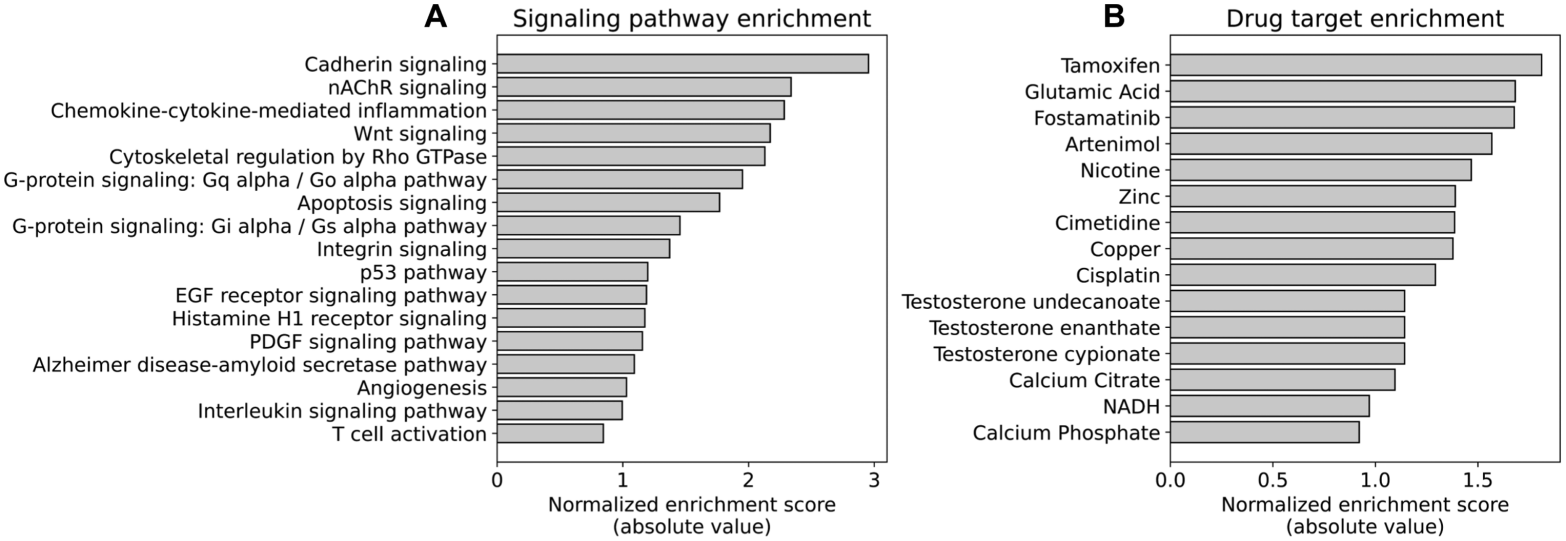
Enrichment of functional gene sets in regions of significant genetic covariance. (**A**) Gene set enrichment analysis of PANTHER pathways among genes in regions of high covariance reveals enrichment of pathways related to cell-cell adhesion and connective tissue development. (**B**) Analysis of drug target gene sets shows enrichment of genes modulated by zinc, copper, and calcium ions in correlated regions, as well as enrichment of targets for tamoxifen.

We next measured functional pathway enrichment among the list of regions of high covariance using gene set enrichment analysis. Regions with high covariance were significantly enriched for signaling pathways involved in cell-cell adhesion and connective tissue maturation, including cadherin signaling, Wnt signaling, and Rho-GTPase–mediated cytoskeletal regulation (Fig. 2A). We also analyzed enrichment among drug-target gene sets for existing pharmacologic agents, with the most enriched compounds including the estrogen receptor modulator tamoxifen, as well as several metal ions involved in extracellular matrix composition, including copper and zinc (Fig. 2B).

## Discussion

Structural corneal dysfunction is associated with significant visual morbidity in corneal ectatic diseases like keratoconus. While corneal biophysical properties are highly heritable, their genomic basis remains poorly understood.^33^ Our findings demonstrate that structural attributes of the human cornea share a common genomic architecture with general anthropometric traits. Both CH and CRF showed significant global genetic correlations with height. We further identified 68 genomic regions with significant levels of local genetic covariance between CRF and height. These regions contained 2874 unique genes, which among them demonstrated enrichment of several functional pathways involved in corneal development, including Wnt/cadherin signaling and metal ion homeostasis. Furthermore, expression of these genes was high in corneal endothelial and epithelial cell types, which have been implicated in a causative pathway for keratoconus.^34^^,^^35^

The observed mild but statistically significant global association of height with CH and CRF suggests that corneal structural development relies on genes involved in connective tissue maturation throughout the body. This result provides a quantitative genomic context for the phenotypic associations observed with several corneal conditions, including keratoconus.^1^^,^^36^^–^^38^ In addition to keratoconus itself, several keratoconus-related endophenotypes are associated with overall body habitus. For instance, one recent study identified a correlation between reduced corneal refractive power and greater body height in a European population.^11^ Other studies have demonstrated associations between keratoconus incidence and both higher BMI and lower body height.^39^^,^^40^ In addition to providing evidence of a common genomic architecture for structural corneal traits and body habitus, local genetic covariance analysis also allows for the identification of genomic regions driving this association. While previous GWAS have identified dozens of regions associated with keratoconus endophenotypes, including genes involved in collagen synthesis,^17^ cell differentiation,^15^^,^^41^ and extracellular matrix formation,^42^ such studies collectively explain less than 20% of known heritability for these traits. This discrepancy is thought to derive from the abundant role of common, low-effect variants in corneal biomechanical development, an ideal setting for genetic correlation analysis.^15^

The genes contained within the 68 regions of high covariance facilitate a variety of processes with experimental or associative links to corneal ectasia. Cadherin signaling, which is broadly involved in connective tissue maturation, represented the single most enriched functional pathway among genes in correlated regions, providing additional evidence for its importance in corneal function specifically. For instance, several cadherin-family proteins, such as cadherin 11 and desmoglein 1, are known biomarkers for keratoconus, and N-cadherin is critical for corneal epithelial cell maturation.^43^^–^^45^ We also identified significant enrichment of Wnt signaling genes, which likewise mediate collagen dysfunction in the corneal epithelium and axial skeletal connective tissue.^46^^,^^47^ Beta-catenin, a central Wnt signaling hub, has been implicated as a potentially pathogenic mechanotransducer in keratoconus, and Wnt-protein sequence variants are associated with keratoconus risk.^48^^,^^49^ Finally, we identified three genes, *NOX4, FNDC3B,* and *ADAMTS17*, present in regions of significant local covariance that also have known associations with both keratoconus and body height. In particular, NOX4 mediates oxidative stress response, which is dysregulated in corneal ectasias, whereas FNDC3B is broadly involved in cellular differentiation.^50^^–^^52^

We also observed enrichment of gene sets interacting with metal cations such as zinc, copper, and calcium and the selective estrogen receptor modulator tamoxifen. Although the therapeutic potential of such compounds in corneal ectasia remains to be established, these results provide additional support for the involvement of metal ion homeostasis and sex hormone signaling in keratoconus.^53^^–^^57^ In particular, metal ion homeostasis, including of zinc, copper, and calcium specifically, is critical for extracellular matrix development and other supportive structures, providing a potential explanation for its shared role in both height and keratoconus endophenotypes.^58^^–^^60^ Such associations may be therapeutically relevant, because a recent Phase 1/2a clinical trial of copper eyedrops showed a significant reduction in corneal steepness in keratoconus patients (iVeena Delivery Systems. Safety and preliminary efficacy of IVMED-80 eye drops in keratoconus patients. ClinicalTrials.gov identifier NCT05241145.)

Our analysis has several limitations. The study population is predominantly of European ancestry, which may limit the generalizability of the results. We also note that although LD regression analysis identifies correlative relationships between genomic landscapes, it does not assess causal relationships, is limited in its ability to infer significance at the individual gene level, and may be susceptible to bias because of phenotype-specific mating patterns.^61^ Although our downstream pathway and expression analyses, as well as a lack of evidence for assortative mating in corneal ectasia, support the biological relevance of our genetic correlation findings, our analysis relies on the standard, imperfect assumptions of genetic correlation analysis. Furthermore, several groups have reported paradoxically inverted directionality findings from global genetic correlation analysis in comparison to corresponding phenotypic associations.^62^^,^^63^ Although the theoretical basis for this phenomenon remains poorly understood, it nonetheless limits our ability to make assumptions about the direction of a phenotypic association based on genetic correlations. Finally, we acknowledge that ρ-HESS is inherently an estimation tool. Other approaches to assess local genetic correlation, such as LOGODetect (which implements a scan statistic search^64^), SUPERGNOVA (which uses a random effects model^62^), and LAVA (which uses partial correlation and multiple regression^65^), could provide additional insights into local correlation patterns. Ultimately, given the associative nature of genetic correlation analysis, knockout and overexpression studies will be necessary to make conclusions about causality.

Corneal structural abnormalities underly ectatic disease and yet remain poorly understood at a genetic level. Our results suggest that corneal development shares a common genomic architecture with gross body habitus, informing the design of both clinical biomarkers and therapeutic interventions. Further investigations, including in vivo interrogation of candidate genes, will provide additional insight into this relationship.

## Supplementary Material

Supplement 1
