# Supplementary material for: Genetic Correlations Among Corneal Biophysical Parameters and Anthropometric Traits
Source: Transl Vis Sci Technol. 2023 Aug 10;12(8):8. doi: 10.1167/tvst.12.8.8 (PMC10424803; doi:10.1167/tvst.12.8.8)
Supplement: Supplement 1 [file tvst-12-8-8_s001.pdf]

SUPPLEMENTARY MATERIAL

Supplementary Table S1. **Power estimates for pairwise global genetic correlation analyses**

	**CH**	**CRF**	**3mmK**	**BMI**	**Weight**	**Height**
**CCT**	9,344	10,447	1,451	16,449	23,096	30,732
**CH**	--	17,135	2,380	26,979	37,881	50,406
**CRF**	--	--	2,661	30,163	42,352	56,355
**3mmK**	--	--	--	4,189	5,882	7,827
**BMI**	--	--	--	--	66,683	88,730
**Weight**	--	--	--	--	--	124,587

[a] Statistical power is estimated as the square root of the product of the first trait heritability, first trait sample size, second trait heritability, and second trait sample size. Abbreviations: CCT: central corneal thickness; CH: corneal hysteresis; CRF: corneal resistance factor; 3mmK: 3mm index of keratometry result; BMI: body mass index; UKBB: UK Biobank

Supplementary Table S2. **Sex-stratified genetic correlations among corneal and anthropometric traits**

	**CCT**	**CH**	**3mmK**	**BMI**	**Weight**	**Height**
**CRF in females only (N=40,769; h2 = 0.27 [0.02])**	0.72 (0.06) p=3.0e-30	0.89 (0.02) p<1e-100	-0.09 (0.22) p=0.68	0.00 (0.03) p=0.97	-0.03 (0.02) p=0.18	-0.11 (0.02) p=6.0e-6
**CRF in males only (N=35,861; h2 = 0.23 [0.03])**	0.67 (0.07) p=7.2e-24	0.86 (0.02) p<1e-100	-0.39 (0.34) p=0.26	0.04 (0.04) p=0.30	0.02 (0.03) p=0.55	-0.11 (0.03) p=6.0e-4

[a] Data are presented as global rg (SE); p-value. Abbreviations: N: sample size; h2: GWAS-explained heritability; CCT: central corneal thickness; CH: corneal hysteresis; CRF: corneal resistance factor; 3mmK: 3mm index of keratometry result; BMI: body mass index; UKBB: UK Biobank

Supplementary Table S3. **List of regions with significant local genetic covariance for corneal resistance factor and height**

chr	start	end	num_snp	local_rhog	var	se	p
3	139954597	141339097	2119	0.0016524	3.04E-08	0.00017449	2.80E-21
5	107264196	108633934	2727	-0.0007632	7.17E-09	8.47E-05	1.97E-19
3	170964909	172295731	2551	-0.0010969	1.69E-08	0.00012985	2.98E-17
21	47492226	48119752	1721	0.00059253	5.21E-09	7.22E-05	2.17E-16
1	88128631	90066303	2633	-0.0006273	6.72E-09	8.20E-05	2.01E-14
2	237449032	238760981	2947	0.00053059	4.93E-09	7.02E-05	4.24E-14
17	9965921	11102815	2304	-0.0004134	3.14E-09	5.60E-05	1.58E-13
17	36809344	38877404	2459	-0.0006299	7.82E-09	8.84E-05	1.04E-12
15	67094767	69017999	3143	-0.0005851	7.01E-09	8.37E-05	2.82E-12
15	100636847	101552694	2295	-0.0007267	1.12E-08	0.00010588	6.73E-12
8	22897057	24674718	3561	0.00069835	1.06E-08	0.00010291	1.15E-11
12	65559695	67181144	2436	-0.0010745	2.53E-08	0.00015906	1.43E-11
3	135456906	137371083	2594	0.00054215	6.59E-09	8.12E-05	2.39E-11
1	148361253	151538881	2073	-0.0008501	1.65E-08	0.00012827	3.41E-11
4	105305294	107501305	3300	0.00060946	8.63E-09	9.29E-05	5.29E-11

2	171226245	173138905	3336	-0.0005444	7.06E-09	8.40E-05	9.20E-11
11	47006137	49866050	5416	0.00057097	7.94E-09	8.91E-05	1.46E-10
9	3190064	4495328	2821	0.00044583	5.05E-09	7.10E-05	3.48E-10
2	242149921	243188920	1618	-0.0004633	5.53E-09	7.44E-05	4.69E-10
11	119215476	120766806	2998	-0.0003922	4.00E-09	6.32E-05	5.56E-10
2	110572432	113921856	3920	-0.0005398	7.61E-09	8.73E-05	6.15E-10
6	125424383	127540461	2174	-0.0007291	1.42E-08	0.00011907	9.17E-10
11	55082657	58457495	8868	0.00050468	6.83E-09	8.26E-05	1.01E-09
5	63968304	65911286	3145	-0.0004543	5.74E-09	7.58E-05	2.01E-09
2	31550450	33363777	2507	-0.0004965	7.37E-09	8.59E-05	7.38E-09
4	7539692	8152235	1856	-0.0003138	3.19E-09	5.65E-05	2.74E-08
11	54695473	55082657	1071	0.00033435	3.73E-09	6.11E-05	4.43E-08
13	75670143	77410555	3496	-0.0002991	3.00E-09	5.48E-05	4.71E-08
20	39610856	40585689	1369	0.00039276	5.35E-09	7.31E-05	7.84E-08
11	89208936	90966490	3041	-0.0003071	3.40E-09	5.83E-05	1.37E-07
5	129519025	132139649	2798	0.00048754	8.74E-09	9.35E-05	1.84E-07
14	59448336	61680424	3383	-0.0005248	1.03E-08	0.00010151	2.34E-07
11	65898631	68005825	2691	-0.000613	1.44E-08	0.00011992	3.20E-07

5	178413464	179401244	2077	-0.0003544	4.84E-09	6.96E-05	3.51E-07
11	49866050	54695473	4996	0.00033635	4.38E-09	6.62E-05	3.68E-07
6	169382050	170330173	2218	0.00031019	3.91E-09	6.25E-05	6.93E-07
4	72140558	74592390	3436	-0.0004551	8.43E-09	9.18E-05	7.19E-07
11	63804569	65898631	2356	-0.0004073	6.86E-09	8.28E-05	8.77E-07
1	5913893	7247335	2346	-0.0002728	3.09E-09	5.55E-05	9.02E-07
1	1892607	3582736	3534	-0.0003654	5.64E-09	7.51E-05	1.14E-06
5	140645971	142981248	3672	-0.0003823	6.20E-09	7.87E-05	1.20E-06
17	59312755	61545589	2442	-0.0006229	1.65E-08	0.00012849	1.25E-06
2	33363777	35345028	4827	-0.000388	6.41E-09	8.01E-05	1.27E-06
9	115693145	117019801	2645	-0.0003373	4.87E-09	6.98E-05	1.35E-06
20	31614823	32813441	1262	-0.0005977	1.54E-08	0.00012422	1.49E-06
7	19481547	20124908	1517	-0.0003462	5.20E-09	7.21E-05	1.56E-06
10	42372579	43894771	3464	-0.0002547	2.83E-09	5.32E-05	1.72E-06
4	183371275	184929455	3838	-0.0002303	2.33E-09	4.83E-05	1.84E-06
5	11940	982252	2082	-0.0002901	3.79E-09	6.16E-05	2.46E-06
16	60054	1207206	2692	-0.0005305	1.28E-08	0.00011311	2.72E-06
18	59020751	60277715	2738	-0.0002523	2.93E-09	5.41E-05	3.09E-06

6	21684065	22748307	2497	0.00027501	3.53E-09	5.94E-05	3.63E-06
1	170557776	173097907	4732	-0.0005977	1.67E-08	0.00012911	3.67E-06
3	131836516	133252173	3026	-0.0002203	2.30E-09	4.79E-05	4.29E-06
9	135298842	137041122	4101	0.00035771	6.06E-09	7.79E-05	4.34E-06
7	23471442	25077259	3666	-0.0004315	8.83E-09	9.39E-05	4.37E-06
2	218395480	220454551	2481	-0.0005319	1.36E-08	0.00011644	4.92E-06
3	144410816	145664619	2874	-0.0001833	1.69E-09	4.12E-05	8.51E-06
1	169086324	170557776	3443	-0.0002461	3.06E-09	5.53E-05	8.64E-06
1	182755356	184595513	3017	-0.0004918	1.23E-08	0.0001109	9.23E-06
16	82747728	83477208	2504	-0.000181	1.67E-09	4.08E-05	9.26E-06
8	41721454	42773823	1001	-0.0002257	2.68E-09	5.17E-05	1.29E-05
19	8347513	9238393	1793	-0.0004114	8.95E-09	9.46E-05	1.37E-05
15	73628714	76398624	3164	-0.0005373	1.58E-08	0.00012589	1.98E-05
1	194107442	196176201	4410	0.00016464	1.52E-09	3.90E-05	2.45E-05
16	63691589	65938566	4273	-0.0001921	2.08E-09	4.56E-05	2.51E-05
7	42001811	43159074	2188	-0.0002426	3.33E-09	5.77E-05	2.65E-05
4	17383322	18841874	2584	-0.0006535	2.44E-08	0.00015625	2.88E-05
